# Case Report: Intestinal Nodular Lymphoid Hyperplasia as First Manifestation of Activated PI3Kδ Syndrome Due to a Novel *PIK3CD* Variant

**DOI:** 10.3389/fped.2021.703056

**Published:** 2021-10-06

**Authors:** Antonio Marzollo, Silvia Bresolin, Davide Colavito, Alice Cani, Paola Gaio, Luca Bosa, Claudia Mescoli, Linda Rossini, Federica Barzaghi, Giorgio Perilongo, Alberta Leon, Alessandra Biffi, Mara Cananzi

**Affiliations:** ^1^Division of Pediatric Hematology, Oncology and Stem Cell Transplant, Padua University Hospital, Padua, Italy; ^2^Fondazione Citta' della Speranza, Istituto di Ricerca Pediatrica, Padua, Italy; ^3^Istituto di Ricerca Pediatrica, Citta' della Speranza, Padua, Italy; ^4^Research and Innovation (R and I Genetics) Srl, Padua, Italy; ^5^Unit of Pediatric Gastroenterology, Digestive Endoscopy, Hepatology and Care of the Child With Liver Transplantation, Department of Women's and Children's Health, University Hospital of Padua, Padua, Italy; ^6^Unit of Surgical Pathology and Cytopathology, Department of Medicine (DIMED), University Hospital of Padua, Padua, Italy; ^7^Pediatric Immunohematology and Stem Cell Program, Istituto di Ricovero e Cura a Carattere Scientifico (IRCCS) San Raffaele Scientific Institute, Milan, Italy

**Keywords:** inborn error of immunity, nodular lymphoid hyperplasia-GIT, activated PI3K-delta syndrome, novel variant, sirolimus, *PIK3CD*

## Abstract

Nodular lymphoid hyperplasia (NLH) is a lymphoproliferative disease caused by non-clonal expansion of lymphoid cells in the gut mucosa. Little is known about the pathogenesis of NLH, which is often disregarded as an insignificant or para-physiologic phenomenon. We present the case of a girl with isolated diffuse NLH (extending from the stomach to the rectum) caused by activated PI3Kδ syndrome (APDS) due to the novel p.Glu525Gly variant in *PIK3CD*. The *gain-of-function* effect of the variant was confirmed by demonstration of over activation of the Akt/mTOR pathway in the patient's cells. APDS diagnosis led to treatment with sirolimus, which resulted in the complete remission of NLH and in the prevention of extra intestinal complications. In conclusion, we identify APDS as a novel cause of isolated NLH and suggest that patients with severe pan-enteric NLH should be screened for this disorder that may not be apparent on first-line immunological testing.

## Introduction

Lymphoid follicles are part of the gut-associated lymphoid tissue (GALT) and can be normally found in the mucosal and submucosal layers of the gastrointestinal tract, where they are mainly involved in immune surveillance and mucosal repair (1). Physiologically, they are predominantly located in the terminal ileum and in the anorectal region, while they are barely represented in the stomach and in the duodenum (1).

Intestinal nodular lymphoid hyperplasia (NLH) is a benign lymphoproliferative disease characterized by a diffuse or focal hyperplasia of lymphoid follicles along the intestine due to an accumulation of nonmalignant lymphoid cells in the gut mucosa (2). Upon endoscopy, NLH is defined as a cluster of ≥10 extruding lymphoid nodules, each at least 2 mm in diameter (3). It is mainly observed in the small intestine, less commonly in the large intestine, and rarely in the stomach or in the duodenum. Histologically, NLH is characterized by the presence of polymorphous hyperplastic lymphoid follicles with highly active germinal centers and well-defined lymphocyte mantles and is confined to the lamina propria and/or the superficial submucosa (2, 4). The exact epidemiology of NLH is unknown as published literature mainly includes case reports and small series of patients. NLH has been observed at any age, but it has most frequently been reported during childhood (4). Clinical manifestations include diarrhea, malabsorption, gastrointestinal bleeding, and abdominal pain, but many affected patients may be asymptomatic, and NLH can be an incidental finding (5, 6). Despite NLH being a non-clonal benign lesion, its presence has been reported as a risk factor for intestinal lymphoma (4). The pathogenesis of NLH is largely unknown, but several conditions have been associated with its development. These include viral (CMV, EBV, and HIV), bacterial (*Helicobacter pylori, Yersinia enterocolitica*), or parasitic (*Giardia lamblia*) infections, cow's milk protein allergy, familial Mediterranean fever (FMF), and other inborn errors of immunity (IEI) (3, 7). Among patients with IEI, NLH has been associated with common variable immune deficiency and selective IgA deficiency (2). To date, no genetically defined disease other than FMF has been shown to cause isolated NHL. We report here the case of a patient with activated PI3 kinase δ syndrome (APDS) due to a novel variant in *PIK3CD* presenting with isolated severe NHL.

## Materials and Methods

### Genetic Analysis

To explore genetic causes of the dysregulation of the intestinal mucosal immune response, whole-exome sequencing was performed with an Agilent® clinical exome research kit and Illumina® sequencing technology, as previously described (8).

### Cell Culture

Human peripheral blood mononuclear cells (PBMCs) were isolated by Ficoll density gradient centrifugation, washed twice in Hank's BSS sterile solution (BioConcept, Allschwil, CH), and resuspended at 1 × 10^6^ cells/ml with complete RPMI-1640 medium (Biochrom AG, Berlin, DE) containing 10% fetal bovine serum (FBS), 2 mM l-glutamine (Life Technologies, Carlsbad, USA), and penicillin/streptomycin antibiotics (100 U/ml, Life Technologies, Carlsbad, USA). Cells were stimulated with 1 μg/ml of anti-CD3 antibody (BD Biosciences, USA) and 100 IU/ml human IL-2 (Cell Guidance Systems, UK). Control cells were cultured without IL-2 and anti-CD3 antibody. After 3 days, stimulated T cells and control cells were washed and collected for lysis.

### Western Blot

Proteins were extracted from activated T cells with T-PER and NaCl 5 M lysis buffer complete with protease and phosphatase inhibitor cocktail (Sigma-Aldrich, Darmstadt, DE). Protein was quantitated by Pierce BSA protein assay kit (Thermo Fisher Scientific, UK). Equal amounts of proteins (20 μg) were resolved using SDS-PAGE gels and transferred to polyvinylidene difluoride (PVDF) Immobilon-p membranes (Merck-Millipore, Darmstadt, DE). Membranes were blocked with I-block™ (Thermo Fisher Scientific, Waltham, MA) for at least 1 h at room temperature and then were incubated overnight at 4°C under constant shaking with the primary antibody against p-Akt Ser 473 (Cell Signaling Technology, IT) or Akt (Cell Signaling Technology, IT). β-Actin (Sigma-Aldrich, Saint Louis, MO) was used as loading control. Membranes were next incubated with horseradish peroxidase (HRP)-conjugated anti-mouse and anti-rabbit antibodies (GE Healthcare, IT). All bands were visualized using ECL Select (GE Healthcare, IT), acquired with a GelDoc 2000 system (Bio-Rad, IT), and quantified by densitometry measurements using the using ImageJ software.

Fold change (FC) of pAkt/Akt was calculated in each sample as the ratio between pAkt and total Akt in stimulated cells and non-stimulated cells. Mean and SEM across three independent experiments were obtained. Multiple *t*-test was used to calculate the statistical difference between pAkt/Akt in stimulated (+) and control (–) cells in each sample.

## Case Description

**Figure 1:**
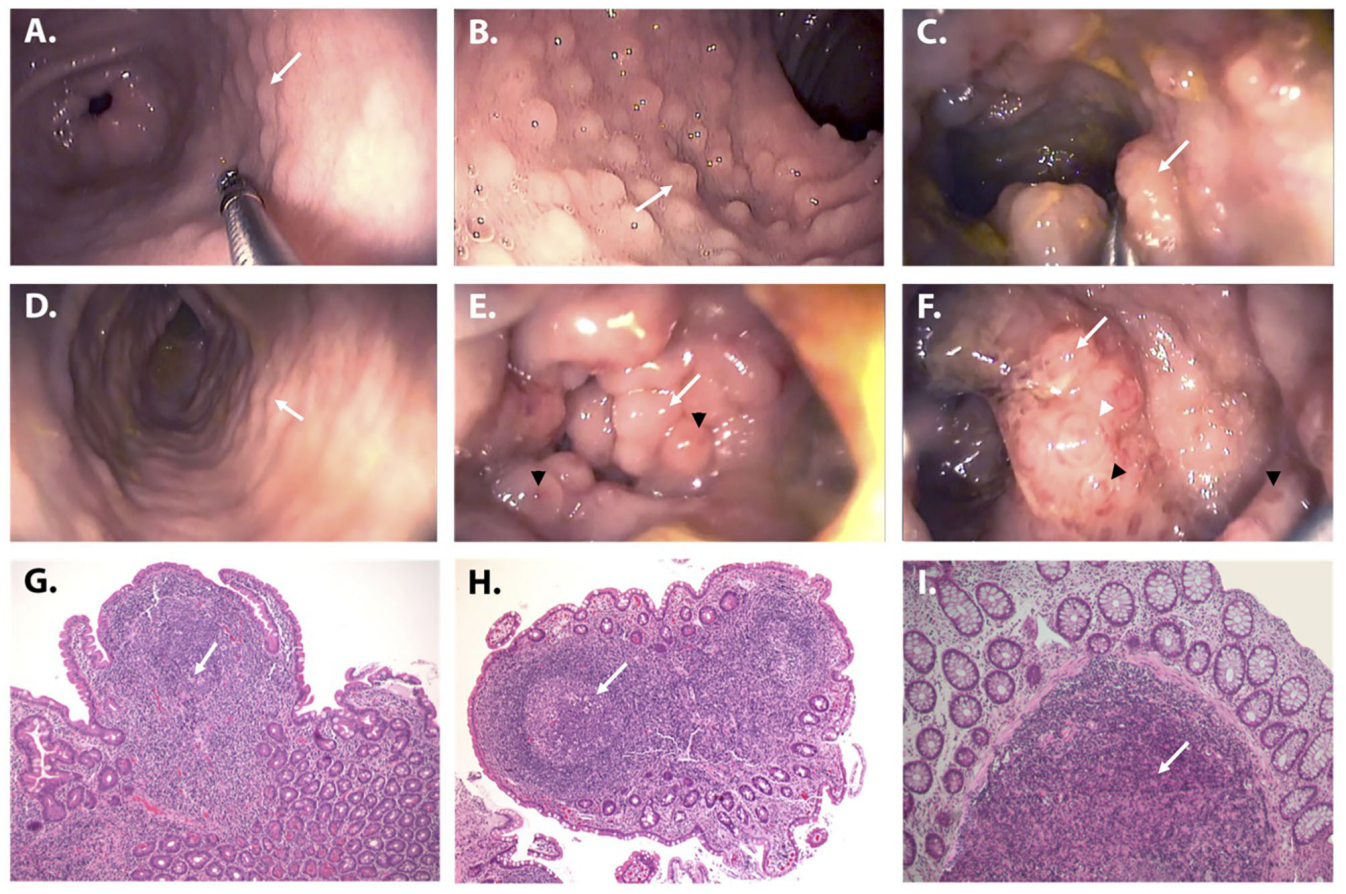
Endoscopic and histologic appearance of NLH in the patient. **(A–F)** Upper and lower gastrointestinal endoscopy demonstrating the presence of multiple nodules (from 0.2 to 1 cm in diameter) throughout the entire gastrointestinal tract (white arrows): stomach **(A)**, duodenum **(B)**, terminal ileum **(C)**, ascending, transverse, and descending colon (representative image), **(D)** sigmoid colon **(E)**, and rectum **(F)**. The nodules were particularly prominent in the recto-sigmoid tract where they had a polypoid appearance, tended to obliterate the lumen, and presented a “red ring sign” around the base (white arrowhead) and mucosal ulcerations on the top (black arrowheads) **(E,F)**. **(G–I)** Histologic examination of the mucosal samples collected along the gastrointestinal tract showed a diffuse hyperplasia of lymphoid follicles (white arrows), consistent with the diagnosis of NLH (H&E stain, ×10). The histologic examination (H&E, ×10) of the mucosal samples collected along the gastrointestinal tract showed a diffuse hyperplasia of lymphoid follicles (white arrows), without inflammation, granulomas, or other histological lesions.

The patient is the only daughter of non-consanguineous parents of Italian origin. At the age of 5 years, she had repeated episodes of hematochezia, which progressively evolved into chronic bloody mucous diarrhea lasting for over 4 weeks (9). When the girl was firstly evaluated at 5.5 years of age, physical examination was normal, and growth was regular. Her family and personal history were unremarkable, without any opportunistic or severe infection. Stool culture for bacteria and stool tests for viruses and parasites were negative. Fecal calprotectin showed repeatedly elevated results (>2,100 μg/g, normal value < 50 μg/g), while C-reactive protein and erythrocyte sedimentation rate were normal. Anti-neutrophil cytoplasmic antibodies (ANCA) and anti-*Saccharomyces cerevisiae* antibodies (ASCA) were negative. Sugar intestinal permeability was markedly altered (lactulose/mannitol ratio 0.09, normal value < 0.03). Upper and lower gastrointestinal digestive endoscopy showed numerous small nodules throughout the entire gastrointestinal tract from the stomach to the rectum (Figures 1A–F), whose histopathologic features were consistent with the diagnosis of NLH (Figures 1G–I). No signs of chronic intestinal inflammation or autoimmune enteropathy, such as enterocyte apoptosis, were observed in the multiple biopsies taken at endoscopy. Known infectious causes of NLH, namely, CMV, EBV, HIV, *Yersinia enterocolitica, Helicobacter pylori*, and *Giardia lamblia* infections, were excluded (3). Familial Mediterranean fever was ruled out by direct sequencing of the *MEFV* gene (7). A complete immunological evaluation was performed, showing substantial elevation of total IgG with normal first-line lymphocyte subset analysis and valid serological response to tetanus and diphtheria toxoids (Table 1). To exclude a cow's milk allergy (10), a trial of cow's milk protein-exclusion diet was attempted without improvement. Subsequently, the patient received a short course of steroids (oral prednisone at an initial dose of 1.5 mg/kg/day) with complete resolution of symptoms and normalization of calprotectin during treatment but rapid clinical and biochemical (i.e., calprotectin elevation) relapse upon discontinuation.

**Table 1:** Evolution of clinical symptoms, fecal calprotectin, and immunological parameters over time.

	**Normal values**	**Age**			
		**5 years 7 months**	**6 years**	**6 years 5 months**	**7 years 6 months**
Treatment	-	None	None	Sirolimus, Ig substitution, azythomycin	Sirolimus, Ig substitution, azythomycin
GI manifestations	-	Bloody mucous diarrhea	Bloody mucous diarrhea	No symptoms	No symptoms
Fecal calprotectin (μg/g)	<50	>2,100	>2,100	365	14
IgG (g/L)	5.52–11.98	16.9 ↑	14.06 ↑	-	-
IgA (g/L)	0.41–2.57	0.46	0.40	0.42	0.52
IgM (g/L)	0.40–1.45	1.46	1.42	0.83	0.98
Anti-tetanus toxoid	>0.50	0.76	0.52	-	-
Anti-diphtheria toxoid	>0.10	0.51	0.24	-	-
Lymphocyte number (× 10^9^/L)	1.2–4.6	2.7	3.09	1.78	1.81
CD3+ (%)	60–78	68	64	79	82
CD4+CD3+ (%)	31–47	44	29	35	39
CD8+CD3+ (%)	16–27	21	32	41	37
CD19+ (%)	13–29	26	17	15	12
CD16+CD56+ (%)	5–16	5	19	5	5
Naïve, CD4+CD45RA+ (% of CD4+ Ly)	61.8–85	-	40 ↓	51 ↓	65
Memory, CD4+CD45RO+ (% of CD4+ Ly)	14.8–37.2	-	60 ↑	48 ↑	35
Naïve, CD8+CD45RA+CD27+ (% of CD8+ Ly)	57–83	-	39 ↓	48 ↓	40↓
TEMRA, CD8+CD45RA+CD27- (% of CD8+ Ly)	0.9–17.9	-	10	5	3.3
CD3+CD25+ (%)	0.9–4.7	-	1.9	3	3.6
Double negative T, CD3+CD4-CD8-TCRαβ+ (% of CD3+ Ly)	<2.5	-	1	1.14	1
Naive B, IgD+CD27- (% of CD19+ Ly)	59.7–88.4	-	78	88	65
Marginal zone B, IgD+CD27+ (% of CD19+ Ly)	3.1–18	-	4	9	10
Memory B, IgD-CD27+ (% of CD19+ Ly)	2.9–17.4	-	13	1.7 ↓	15
Transitional B, IgM+CD38+ (% of CD19+ Ly)	9–24	-	48 ↑↑	33 ↑	22
Plasmoblasts, IgM-CD38++ (% of CD19+ Ly)	0.1–3	-	13 ↑	1.7	2.9
Proliferative response (cpm)					
PHA	42.3–426 x 10^3^	-	30.9 ↓	-	-
antiCD3	9.3–203 x 10^3^	-	4.9 ↓	-	-
Tetanus toxoid	0.82–90 x 10^3^	-	1.31 ^*^	-	-
Candida	0.9–51.4 x 10^3^	-	0.14 ↓	-	-
Varicella Zoster Virus	5.8–132 x 10^3^	-	0.44 ↓	-	-

* *: despite a normal absolute number, this value was deemed below normal due to a high spontaneous proliferation of cells (2.6 × 10^3^ cpm), resulting in a stimulation index of 0.5*.

**Figure 2:**
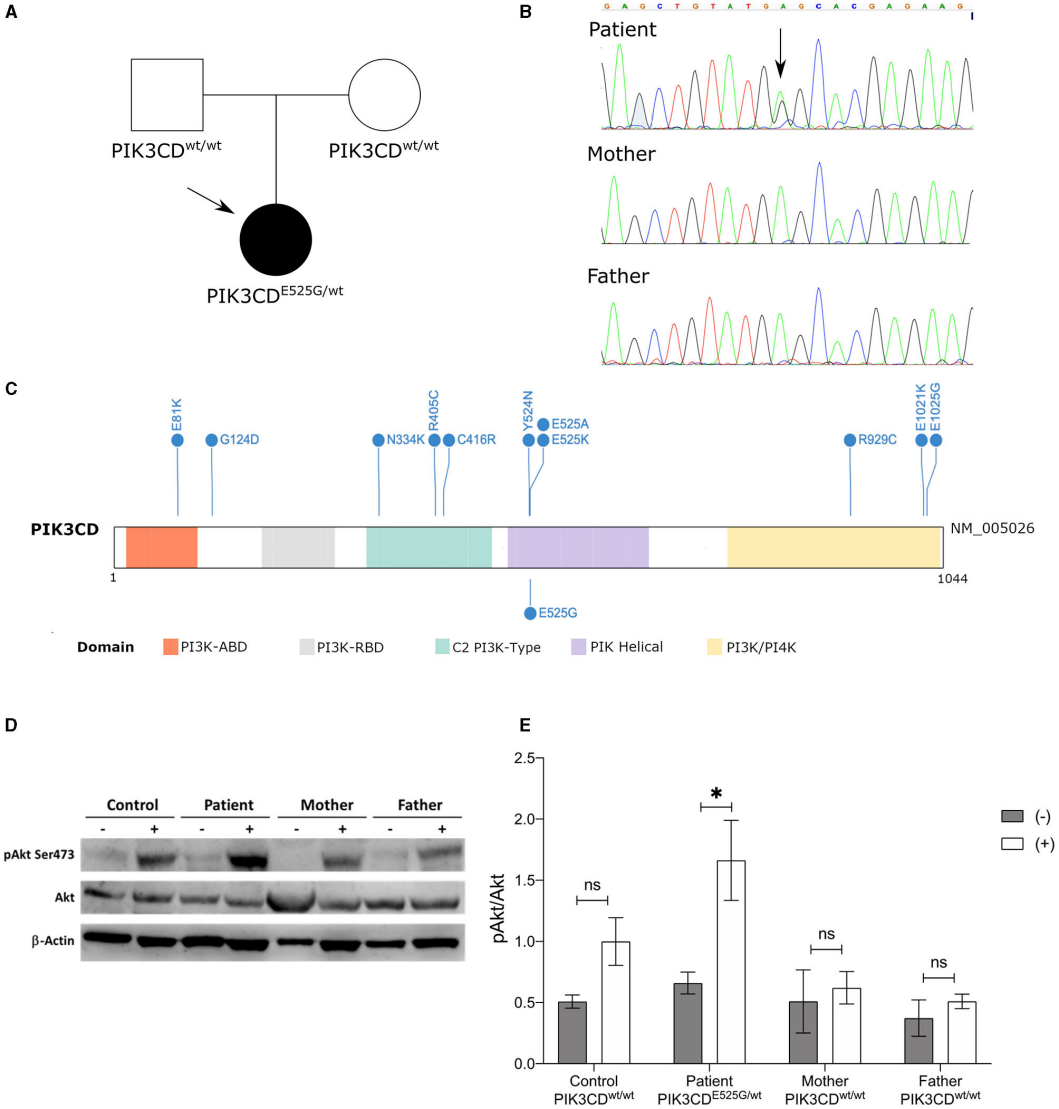
Genetic and functional characterization. **(A)** Pedigree of the family indicating the *de novo* occurrence of the variant. **(B)** Sanger electropherograms. **(C)** *PIK3CD* protein domain plot showing known activating variants (above) (15) and the variant described here (below). **(D)** Immunoblot of stimulated (+) and non-stimulated (–) T cells derived from PBMCs collected from the patient, her mother and father, and a normal control (CTRL). Expression of Akt and p-Akt (S473) is shown. β-Actin was employed as loading control. **(E)** Fold change of phosphorylation of p-Akt (S473)/Akt in activated T cells (+) and non-activated cells (–) from the patient, parents, and healthy control. Bars are represented as mean ± SEM. *p < 0.05; ns: not significant.

The observation of severe pan-enteric NLH in a young child together with the exclusion of intestinal infections and the presence of chronic-recurring symptoms elicited the diagnostic suspicion of an inherited immunological defect despite normal first-line tests. Given the absence of a guiding-diagnostic element, a high-throughput genetic test was performed to explore genetic causes of immune dysregulation and immune deficiency. Whole-exome sequencing with phenotype-driven analysis was performed, focusing on genes primarily implicated in immunological diseases (11, 12), revealing the presence of the heterozygous variant p.Glu525Gly (c.1574A>G) in the *PIK3CD* gene. Sanger sequencing confirmed the presence of this variant, and segregation analysis demonstrated its *de novo* occurrence (Figures 2A,B). The variation affects the same codon of two previously reported *PIK3CD* variants causing APDS, namely, c.1573G>A, p.(Glu525Lys) and c.1573A>C, p.(Glu525Ala) (Figure 2C) (13, 14). It has not been identified previously, and it is absent from the largest allele frequency databases (gnomAD, EVS, and 1000 Genomes Project). PolyPhen-2, SIFT, CADD-Phred, and MutationTaster prediction algorithms indicate with high confidence a deleterious effect on the resulting protein. Conservation tools, such as phyloP, GERP, and PhastCons, indicate that the DNA region harboring the variant is highly evolutionary conserved.

To confirm the pathogenic role of the p.Glu525Gly variant, extended immunological phenotyping was performed along with *in vitro* functional tests. Detailed immune subset analysis demonstrated reduced CD4+CD45RA+ and CD8+CD45RA+CD27+ naïve T helper cells and elevated CD19+IgM+CD38+ transitional B cells (Table 1). T-cell proliferation was impaired upon stimulation with mitogens (PHA and anti-CD3), tetanus toxoid, *Candida*, and varicella zoster virus antigens. Given that PI3Kδ-activating mutations responsible for APDS induce an over activation of the Akt/mTOR pathway (13, 16), the quantity and the activation of AKT (pAkt-Ser473) were tested in the patient, in her parents (none of them carrying the p.Glu525Gly variant), and in a healthy control. After PBMC stimulation with anti-CD3 antibody and IL-2, significant hyperphosphorylation in the S473 residue of the AKT was observed in the patient as compared to her parents and the healthy control (Figures 2D,E), proving that the p.Glu525Gly variant behaves as a *gain-of-function* and causes PI3Kδ hyperactivation (13). Thus, the girl received a diagnosis of APDS.

In light of this, notwithstanding the absence of respiratory symptoms, a chest CT scan was performed, showing multiple small nodules in the lung parenchyma and mediastinal lymphadenopathies. Treatment with sirolimus (initial dose 2 mg/m^2^ aiming at levels of 4–12 ng/ml, with an optimal target of 9 ng/ml) was then initiated, leading to the gradual resolution of gastrointestinal symptoms and to the normalization of fecal calprotectin in one year (Table 1). Due to the occurrence of few episodes of respiratory infection, including one episode of pneumonia and the notion of a disturbed immunoglobulin efficacy despite normal levels in APDS patients (17), immunoglobulin substitution and azithromycin prophylaxis (10 mg/kg/day, 3 days/week) were started with prompt interruption of recurrent infections. At the last evaluation, the patient was 7.5 years old and asymptomatic. During the 18 months of treatment, she did not experience any clinically relevant infection or autoimmune disorder. Moreover, the numbers of naïve T cells and transitional B cells returned to normal values (Table 1). At the age of 7.5 years, the patient was diagnosed with childhood absence epilepsy, which was deemed unrelated to APDS, based on the absence of an association of the two disorders in the literature. Brain magnetic resonance imaging at the onset of epilepsy was unremarkable.

## Discussion

APDS (OMIM # 615513) is an inborn error of immunity caused by autosomal dominant *gain-of-function* variants in the *PIK3CD* or *PIK3R1* genes, which encode for the catalytic and regulatory subunits of the phosphoinositide 3-kinase δ complex (13, 16, 18). The variants underlying APDS cause hyperactivation of the PI3K/AKT/mTOR/S6 kinase signaling cascade, which controls cell growth, proliferation, and metabolism (19, 20). Since PIK3CD and PIK3R1 are mainly expressed in lymphocytes, the most prominent physiological alterations of this condition are found in the immune system. APDS causes enhanced T-cell senescence with reduced naïve T cells and impaired B-cell responses (21). Follicular helper T cells (Tfh) are increased, resulting in the expansion of germinal centers in peripheral lymphoid organs (22). These immunological mechanisms underlie the clinical phenotype of patients with APDS, characterized by recurrent bacterial and viral infections, autoimmune disorders, severe lymphadenopathy, and increased risk of lymphoma, similar to other IEIs associated with immune dysregulation (23, 24). Gastrointestinal manifestations are reported in up to 50% of patients and include a broad spectrum of disorders (colitis, autoimmune enteropathy, and NLH) (17, 25). However, gastrointestinal involvement is typically not isolated and usually occurs later in the course of the disease (15).

APDS is usually suspected when patients develop recurrent pulmonary infections, lymphadenopathy, or hepatosplenomegaly in their first decade of life, and not in patients with isolated NLH. The diagnosis of APDS can be challenging, since first-level immunological tests (lymphocyte count, immunoglobulin levels, and basic lymphocyte subsets) can be normal, and gastroenterologists may have limited knowledge of recently identified IEIs. The diagnosis of APDS is further hurdled by the need to functionally prove the *gain-of-function* effect of novel variants in *PIK3CD* since it cannot be predicted by computational methods alone. Indeed, each novel variant in *PIK3CD* requires a specific functional validation to confirm its pathogenicity (26). Our report further extends the list of variants associated with this disorder and may be valuable for centers without access to functional testing with a rapid turnaround time.

The recognition of APDS as a cause of NLH had important consequences on the management of our patient. First, it allowed the establishment of targeted therapy for APDS (i.e., sirolimus), which, even if not usually employed for the treatment of NLH, led to the complete resolution of gastrointestinal symptoms (25). Second, it prompted us to investigate and detect the extraintestinal complications of the disease (i.e., subclinical pulmonary involvement). Third, it allowed the initiation of immunoglobulin substitution and azithromycin administration to prevent respiratory infections and bronchiectasis, which are a frequent complication of APDS (17). The diagnosis of APDS will also provide a future benefit for the patient as it will expand the therapeutic options when novel specific treatments for APDS (e.g., specific PI3K inhibitors such as leniolisib or seletalisib) become available (27, 28).

In conclusion, the presence of isolated NLH, especially if severe, widespread through the gastrointestinal tract or pan-enteric, should prompt the suspicion of an inborn error of immunity such as APDS, even in the absence of other clinical signs of immune deficiency and with normal first-line immunological testing. In such circumstances, broad immunological investigations, including multigene panel analysis, should be performed to warrant the prompt institution of a targeted potentially life-saving treatment.

## Data Availability Statement

The data presented in the study are deposited in the ClinVar repository, accession number SCV001810147.1

## Ethics Statement

Ethical review and approval was not required for the study on human participants in accordance with local legislation and institutional requirements. Written informed consent to participate in this study was provided by the participants or their legal guardian/next of kin. Written informed consent was obtained from the participants or their legal guardian/next of kin for the publication of any potentially identifiable images or data included in this article.

## Author Contributions

AM, PG, LB, LR, FB, AB, GP, and MC provided direct clinical care for the patient. SB and AC performed functional testing on the *PIK3CD* variant. DC and AL performed whole exome sequencing and variant interpretation. CM performed histopathological examination. AM, SB, and MC wrote the manuscript. All authors reviewed and approved the manuscript.

## Funding

The work was funded by a grant from Fondazione Città della Speranza ONLUS (http://cittadellasperanza.org/), Associazione di Promozione Sociale Genitori in fuga (https://www.genitoriinfuga.org/), and Associazione sportiva dilettantistica NCO Crew to AM and SB.

## Conflict of Interest

The authors declare that the research was conducted in the absence of any commercial or financial relationships that could be construed as a potential conflict of interest.

## Publisher's Note

All claims expressed in this article are solely those of the authors and do not necessarily represent those of their affiliated organizations, or those of the publisher, the editors and the reviewers. Any product that may be evaluated in this article, or claim that may be made by its manufacturer, is not guaranteed or endorsed by the publisher.
